# The Mesenchymal Cap of the Atrial Septum and Atrial and Atrioventricular Septation

**DOI:** 10.3390/jcdd7040050

**Published:** 2020-11-04

**Authors:** Ray Deepe, Emily Fitzgerald, Renélyn Wolters, Jenna Drummond, Karen De Guzman, Maurice J.B. van den Hoff, Andy Wessels

**Affiliations:** 1 Department of Regenerative Medicine and Cell Biology, Medical University of South Carolina, 173 Ashley Avenue, Charleston, SC 29425, USA; deepe@musc.edu (R.D.); emilyfitz15@gmail.com (E.F.); woltersr@musc.edu (R.W.); drummonj@musc.edu (J.D.); kadeguz@g.clemson.edu (K.D.G.); 2 Amsterdam UMC, Academic Medical Center, Department of Medical Biology, Meibergdreef 15, 1105AZ Amsterdam, The Netherlands; m.j.vandenhoff@amsterdamumc.nl

**Keywords:** atrial septum, valve, dorsal mesenchymal protrusion, cushion, mesenchymal cap

## Abstract

In this publication, dedicated to Professor Robert H. Anderson and his contributions to the field of cardiac development, anatomy, and congenital heart disease, we will review some of our earlier collaborative studies. The focus of this paper is on our work on the development of the atrioventricular mesenchymal complex, studies in which Professor Anderson has played a significant role. We will revisit a number of events relevant to atrial and atrioventricular septation and present new data on the development of the mesenchymal cap of the atrial septum, a component of the atrioventricular mesenchymal complex which, thus far, has received only moderate attention.

## 1. Introduction

The development of a fully septated four-chambered heart is a complex process that involves the contribution of multiple cell populations with distinct embryonic origins, a myriad of regulatory pathways, and a series of complicated spatiotemporal remodeling events. It is crucial to the proper function of the heart after birth that valves and septa form correctly to ensure that the oxygenated and deoxygenated blood are properly separated within the heart. This guarantees that during each cardiac cycle, the blood is propagated unidirectionally through its respective components. Separating the oxygen-rich and oxygen-depleted blood is achieved by the dividing walls between the left and right parts of the heart. The septum between the left and right atrium is the atrial septum, while the septum between the two ventricles is the ventricular septum. The unidirectional flow of blood is controlled by one-way atrioventricular (AV) valves that allow antegrade flow from atria to ventricles but prevent retrograde flow from ventricles into the atria. The process in which septa and valves are formed is commonly referred to as valvuloseptal morphogenesis. In earlier papers, we have described how the AV mesenchymal complex plays a central and critical role in this process [1,2,3,4,5]. The AV mesenchymal complex consists of the AV cushions, the dorsal mesenchymal protrusion (DMP), and the mesenchymal cap on the leading edge of the primary atrial septum (pAS) [4]. While the development of the AV cushions and DMP has been described in detail over the years, the mesenchymal cap has not received much attention.

## 2. Early Heart Development

**Figure 1:**
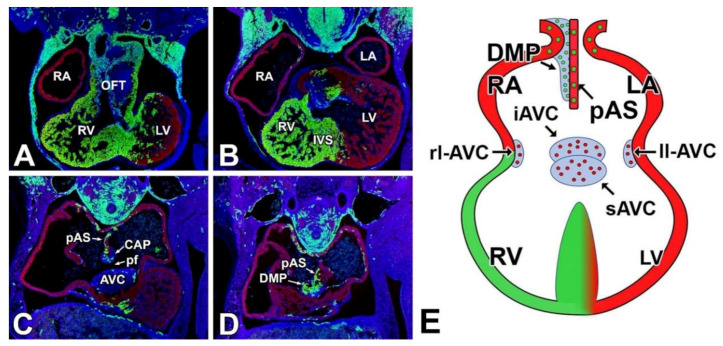
Contribution of First and Second Heart Field to the Developing Heart. Panels (**A**–**D**) show serial sections of an 11ED Mef2c-AHF-cre; ROSA^mT/mG^ mouse heart. All myocardial structures express cardiac Actin (red); the Second Heart Field derived tissues are labeled in green. DAPI staining (blue) was used to identify all nuclei. Panel (**E**) is a schematic representation of an embryonic heart around this stage. Note that the complex three-dimensional structure cannot be properly depicted. DMP—dorsal mesenchymal protrusion; iAVC—inferior atrioventricular cushion; pAS—primary atrial septum; pASD—primum atrial septal defect; pf—primary foramen; LA—left atrium; ll-AVC—left lateral atrioventricular cushion; LV—left ventricle; OFT—outflow tract; rl-AVC—right lateral atrioventricular cushions; RA—right atrium; RV—right ventricle; sAVC—superior atrioventricular cushion; VSD—ventricular septal defect; sAS—secondary atrial septum.

In their studies on the initial stages of the development of the heart, Rawles [6] and Rosenquist and DeHaan [7] illustrated that one of the first steps in this process is the formation of two bilateral heart-forming fields of embryonic mesoderm during gastrulation. This population of “precardiac mesoderm” is now known as the First Heart Field (FHF). The two heart-forming fields eventually fuse at the embryonic midline to form the primary heart tube. After its formation, this heart tube consists of an outer layer of myocardial cells and an inner layer of endocardial cells. An acellular, extracellular matrix-rich substance, commonly referred to as the cardiac jelly, is located between these two cell layers. Roughly 20 years ago, a series of studies revealed the contribution of a second wave of mesodermally derived cardiac precursors to the developing heart. This population of cells is known as the Second Heart Field (SHF) [8,9,10,11,12]. Cell fate tracing studies have established that each heart field contributes to specific compartments of the heart. The left ventricle (LV) and both atria (LA and RA, respectively) are mainly derived from the FHF. Initial studies on the contribution of the SHF to the developing heart mainly focused on its importance for the expansion of the arterial pole, showing that the anterior SHF (aSHF) primarily contributes to the outflow tract, right ventricle, and ventricular septum [9,12,13]. Subsequent studies demonstrated that the posterior SHF (pSHF) plays a role in the development of the venous pole by contributing to the DMP and its derivatives [4,14], the pAS [15], and to the atrial myocardium (Figure 1), although the extent to which the pSHF contributes to the atrial myocardial tissues is still a matter of debate [16].

## 3. Cardiac Mesenchyme and Its Contribution to Cardiac Septation

As described above, the myocardium and endocardium are the first cardiac cell lineages to appear as the primary heart tube develops. Subsequent events in heart development, including cardiac septation, critically rely on the contribution of various populations of mesenchymal cells. Below, we have summarized the most relevant information regarding the origin of these cells as it relates to septation.

### 3.1. Endocardially Derived Mesenchyme

The first mesenchymal cells to appear in the developing heart derive from the endocardium. As the primary heart tube is growing and begins to loop [17,18], the extracellular matrix starts to accumulate in the cardiac jelly underneath the endocardium. This takes place in the outflow tract (OFT) and AV junction. In the OFT, this results in the formation of two endocardial ridges, whereas in the AV junction, it leads to the development of, initially, two AV cushions [19]. Subsequent to the development of these structures, the endocardium lining them goes through a process of endocardial-to-mesenchymal transformation (endMT), in which a subset of the endocardial cells differentiate within the endocardial lining and eventually, bud as endocardially derived mesenchymal cells (ENDCs) which migrate into the underlying ECM-rich space, where they eventually become valve interstitial cells (VICs) [20,21]. This process of endMT has been studied in detail for years and has been demonstrated to be controlled by multiple gene regulatory pathways including the TGFbeta, BMP, Notch, and Hippo signaling pathways [22,23,24,25,26]. Slightly later in development, two additional small AV cushions develop on the lateral walls of the AV canal [19,27]. These lateral AV cushions will also become populated by ENDCs as a result of endMT. It is interesting to note that very little work has been done on the question of how the spatiotemporal development of these two sets of cushions is regulated. In addition to being the source of mesenchymal cells in the developing AV cushions and OFT ridges, the endocardium also gives rise to cells that are found in the mesenchymal cap [4,28,29,30]. Compared to the significant body of work published on the mechanisms that control the development of the AV cushions and OFT ridges, very little is known about the mechanisms that regulate the formation of the mesenchymal cap, about the specific characteristics of the cells that comprise the mesenchymal cap, and about the role of the mesenchymal cap in atrial/atrioventricular septation. Below, we will address the mesenchymal cap in more detail.

### 3.2. Cardiac Neural Crest-Derived Mesenchyme

The neural crest plays a very important part in embryonic development [31]. Cells that derive from the segment of the neural crest located between the mid-otic placode and the third somite are particularly important for heart formation. The work on these so-called “cardiac neural crest-derived cells” (CNDCs) was pioneered by Dr. Margaret Kirby and colleagues. Their studies demonstrated how CNDCs contribute to the developing OFT. Moreover, they showed how perturbation of cardiac neural crest development can lead to the kind of OFT abnormalities seen in patients with congenital heart disease (CHD) [32,33,34,35,36,37,38]. As, to the best of our knowledge, the CNDCs do not play a discernable role in atrial septation, they will not be further discussed in the remainder of this contribution.

### 3.3. Epicardially Derived Mesenchyme

The epicardium is an epithelium located on the surface of the heart. The formation of the epicardium is initiated when cells from the proepicardium attach to the myocardial surface of the looping heart and spread out to form an epicardial sheet. The proepicardium is a conglomerate of mesothelially derived cells that form a cauliflower-shaped structure at the interface between liver and sinus venosus [39,40,41,42,43]. A subset of epicardial cells will undergo an epicardial-to-mesenchymal transformation (epiMT), leading to the formation of epicardially derived cells (EPDCs) [44]. These EPDCs migrate into the subepicardial space located between the epicardium and myocardium in a process similar to that of endMT in the AV cushions (see above). However, unlike the ENDCs, which typically do not migrate into the myocardium, EPDCs have the ability to enter the myocardial walls of the developing heart where they differentiate to interstitial fibroblasts, pericytes, coronary smooth muscle cells, and coronary endothelium [44,45,46,47,48,49]. EPDCs also play an important role in the formation of the annulus fibrosis, separating the working myocardium of atria and ventricles, and contribute significantly to the formation of leaflets of the AV valves where they eventually, just like the endocardially derived cells, turn into VICs [48,50,51]. Like CNDCs (see above), EPDCs do not seem to have an active role in atrial septation.

### 3.4. Second Heart Field-Derived Cells

Cell fate tracing using the SHF-specific Mef2c-AHF-cre mouse [12], as well as staining for the expression of ISL1, a transcription factor characteristically expressed in cells of the SHF [11], have convincingly demonstrated that the mesenchyme of the DMP, an important component of the atrioventricular mesenchymal complex, is derived from the pSHF [4,14]. The DMP develops between ED9.5 and ED10.5 when the pSHF cell population located between the venous pole of the heart (sinus venosus/common atrium) and the foregut expands as a result of proliferation and, using the dorsal mesocardium as a portal of entry, protrudes into the common atrium [1,3]. It is important to point out that there has been some discussion regarding the nomenclature of the structure that we call the DMP. In his studies on the anatomy of human embryos in 1880, the Swiss-born German embryologist Wilhelm His described a “fibrous/mesenchymal” structure within the heart as the “spina vestibuli” [52,53]. Given the original description by His, it is possible that the tissue that we have dubbed the DMP was indeed part of this “spina”. The histological and microscopical techniques at the end of the 19th century were obviously not as advanced as they are today and did not allow for the discrimination between the mesenchymal cells of the DMP, the AV cushions, and the mesenchymal cap. We and others have shown, using molecular and cell fate mapping techniques, that there are in fact distinct differences between the origin and fate of the DMP and the other mesenchymal structures in the atrioventricular region [14,28,54,55]. We therefore believe that the term DMP more accurately reflects the fact that this SHF-derived structure is a unique entity that plays its own, and very specific, role in atrial and AV septation. With that being said, we acknowledge that, for all intents and purposes, the term “spina vestibuli” or “vestibular spine” has been correctly used in the literature over the last 20 years to describe the DMP, for instance, in a number of studies on the relation of the DMP/vestibular spine and congenital heart defects [56,57].

## 4. Atrioventricular Septal Defects—The AV Cushions and the DMP

**Figure 2:**
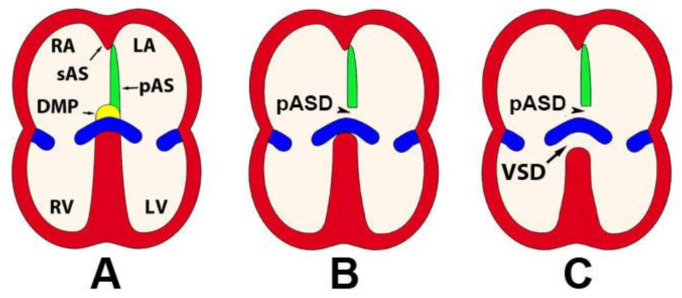
Atrioventricular Septal Defects (AVSDs). Panels (**A**–**C**) show cartoons of a normal heart (**A**), a heart with a partial AVSD (**B**), and a heart with a complete AVSD (**C**). DMP—dorsal mesenchymal protrusion; pAS—primary atrial septum; pASD—primum atrial septal defect; LA—left atrium; LV—left ventricle; RA—right atrium; RV—right ventricle; VSD—ventricular septal defect; sAS—secondary atrial septum.

The mesenchymal tissues described above all play their own specific role in the events that lead to the development of a four-chambered heart with functional AV and OFT valves and properly formed septa. In our contribution to this Special Issue, we will focus on the remodeling events at the AV junction. A number of years ago, the mechanisms that lead to the formation of the AV septal complex gained renewed attention as a result of the emerging insight into the contribution of the DMP to the AV mesenchymal complex and the notion that the DMP could be involved in the pathogenesis of atrioventricular septal defects (AVSDs). AVSDs are serious congenital heart defects found in approximately 5% of all persons born with congenital heart disease (CHD) and a common component of genetic disorders such as Down Syndrome (DS), CHARGE Syndrome (CS), Di George Syndrome [58], and Heterotaxy Syndrome (HS) [59,60,61]. It is important to note that both major forms of AVSDs, i.e., partial AVSD (pAVSDs, Figure 2B) and complete AVSD (cAVSD, Figure 2C), are characterized by the presence of a primary atrial septal defect (pASD or ASD-I), also known as a “ostium primum” defect [52], and a common AV valve (cAVV). In addition, in a cAVSD, a ventricular septal defect (VSD) is also found (Figure 2).

For many years, it was generally believed that AVSDs were exclusively the result of failure of the proper development of the AV cushions. This belief led to the introduction of the term “endocardial cushion defect” [62,63,64,65]. This terminology is still widely used in the medical field as well as in websites that are designed to provide information to the general public about congenital heart disease. Several years ago, however, based on the growing insight into the anatomical, cellular, and molecular mechanisms controlling heart development, it became increasingly clear that mere failure of normal AV cushion development could not account for the complex malformations observed in patients with AVSDs [66]. In particular, abnormal AV cushion formation and/or fusion of the cushions did not seem to satisfactorily explain the pathogenesis of pASD and the presence of cAVV. Among the factors that led to the reconsideration of the role of the endocardial cushions in AVSD pathogenesis was a paper on BMP signaling in valvuloseptal morphogenesis by Jiao and colleagues [67]. In this paper, the importance of BMP4 in heart formation was investigated using a mouse model carrying a hypomorphic BMP4 allele. Analysis of the offspring revealed the presence of heart defects, including AVSDs. Given the prevailing model for the pathogenesis of these defects, it was suggested that the presence of AVSDs was likely the result of perturbation of AV cushion development. However, when we conducted a study in our lab designed to determine the role of BMP signaling in the formation of the AV mesenchymal complex, our in situ hybridization experiments did not show significant levels of BMP4 mRNA in the AV cushions or in the myocardium adjacent to the cushions, but instead we found significant expression of BMP4 mRNA in the mesocardial reflections of the dorsal mesocardium which flank the DMP during its expansion into the common atrium [3]. This observation strongly suggested to us that the role of BMP4 in regulating AV cushion development was likely very limited and that the observed AVSDs in the BMP4 hypomorphic model should likely be attributed to perturbation of the development of other components of the AV mesenchymal complex [3]. Combined, these observations led to the hypothesis that BMP signaling might be an important mechanism controlling the formation of the DMP [3]. To test this hypothesis, the BMP receptor BMPR1A/ALK3 was deleted from the SHF. This approach led to inhibition of the expansion of the pSHF as a result of reduced proliferation, perturbation of DMP development, and eventually resulted in pASVDs [2,3]. Using similar approaches, the importance for Hedgehog signaling in SHF and DMP development was established [1,68,69,70], while other studies showed the importance of Wnt signaling in the development of the DMP [71]. Combined, these results have led to a significant paradigm shift in our understanding of the pathogenesis of AVSDs. While the AV cushions can still be involved in certain aspects of AVSD pathogenesis, the fact that perturbation of SHF development, systematically leads to pASDs with cAVV (i.e., the common defects in all forms of AVSDs) shows that the SHF-derived DMP is a critical component of the AV septal complex and that perturbation of its development is associated with AVSD pathogenesis [72].

## 5. The Mesenchymal Cap

**Figure 3:**
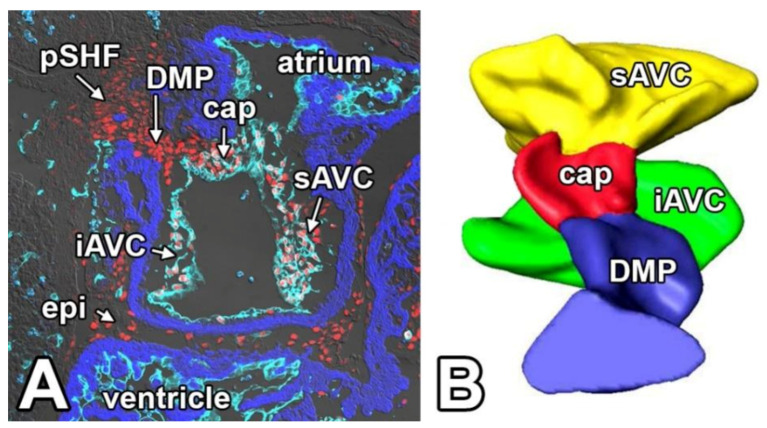
The spatial relationship of the components of the Atrioventricular Mesenchymal Complex at ED11.5. Panel (**A**) shows a sagittal section of an 11.5ED Nfatc1-cre; ROSA^mT/mG^ mouse heart. The endocardial/endocardially derived cells are shown in light turquoise, the myocardial tissues in dark blue, and the expression of the transcription factor SOX9 in red. The image clearly shows how the mesenchyme of the SHF-derived DMP is contiguous with the mesenchymal cap and the inferior AV cushion. In panel (**B**), this situation is schematically depicted. This reconstruction was made in 2007 before we had established that the DMP was derived from the SHF. cap—mesenchymal cap; DMP—dorsal mesenchymal protrusion; epi—epicardium; iAVC—inferior atrioventricular cushion; sAVC—superior atrioventricular cushion; pSHF—posterior second heart field.

While the AV cushions and the DMP have been studied in detail in the context of AV septation, the third component of the AV mesenchymal complex, the mesenchymal cap, has thus far by and large been ignored. In particular, whether mesenchymal cap development plays any role in the context of the pathogenesis of congenital malformations involving the atrial septum and/or the AV junctional complex has not received any serious attention. In fact, the mesenchymal cap is typically only mentioned as functioning as the “glue” that allows the descending primary atrial septum to attach to the AV cushions in the process in which the primary foramen is closed (see below). As we have previously shown [14], the mesenchymal cap and the mesenchyme of the DMP are in continuity with each other. This is also clearly demonstrated in Figure 3. Taking the above into consideration, and given the fact that there are a number of papers that suggest that the mesenchymal cap seems to be abnormal in appearance in the mouse models studied, we have started to develop an interest in investigating the role of the mesenchymal cap in atrial septation. This is done in an attempt to determine whether, and if so how, the cap might play a role in the pathogenesis of defects involving the AV mesenchymal complex. The information on the developing cap in this contribution is not meant to give a comprehensive description, but rather provide a framework for upcoming studies. We will restrict ourselves to a descriptive narrative of some of the features of the cap as they may be relevant in the context of future studies of atrial septation.

## 6. Atrial Septation

**Figure 4:**
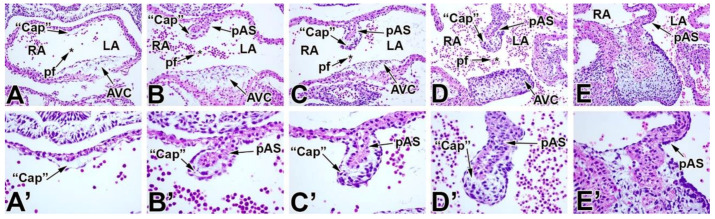
The development of the primary atrial septum and its associated mesenchymal cap in the mouse. The primary atrial septum (pAS) emerges between ED9.5 (**A**,**A’**) and ED10.5 (**B**,**B’**). A virtual acellular cap (Cap) can be observed at ED9.5 (**A**,**A’**). As the pAS continues to grow between ED11.5 (**C**,**C’**) and ED12.5 (**D**,**D’**), the mesenchymal cap also increases in size and the number of mesenchymal cells within the mesenchymal cap is increasing accordingly. At ED 13.5 (**E,E’**) the Cap has fused with the other mesenchymal tissues. FO—foramen ovale; IVS—interventricular septum; LA—left atrium; pAS—primary atrial septum; RA—right atrium; RV—right ventricle.

**Figure 5:**
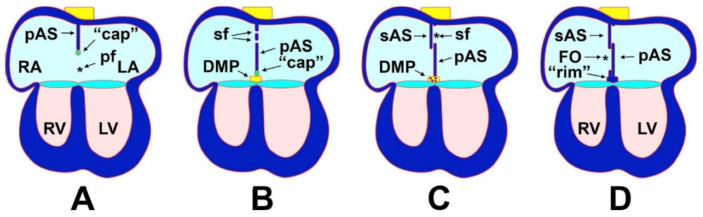
Atrial septation. These simplified diagrams show the critical stages in atrial septation. A detailed explanation is provided in the text. The yellow box in the back of the heart represents the posterior Second Heart Field giving rise to the DMP which in turn develops into the base of the atrial septal complexand becomes muscularized to form the muscular rim (**B**–**D**). The asterisk in (**A**) marks the primary foramen; the asterisk in (**C**) marks the secondary foramen. cap—mesenchymal cap; DMP—dorsal mesenchymal protrusion; FO—foramen ovale; IVS—interventricular septum; LA—left atrium; LV—left ventricle; pAS—primary atrial septum; pf—primary foramen; RA—right atrium; RV—right ventricle; rim—myocardial rim at base of atrial septum; sAS—secondary atrial septum; sf—secondary foramen (from: Burns et al., J. Cardiovasc. Dev. Dis. 2016) [72].

Atrial septation is the process in which the embryonic common atrium becomes physically and functionally divided into a left and right atrium. In this process, a number of different tissues are involved, and several remodeling events take place at the same time. As a result, any description of the complex spatiotemporal process of septation is inherently an oversimplification. In this paper, we will focus on the development of the atrial septum as it is observed in the developing heart of the mouse. It is important to point out that this process in mice is very similar to that seen in humans [28]. Atrial septation (Figure 4 and Figure 5) starts with the formation of the primary atrial septum (pAS—or septum primum). In the mouse, the pAS emerges from the roof of the common atrium between embryonic day 9.5 (ED9.5) (Figure 4A,A’) and ED 10.5 (Figure 4B,B’), in the human at around 4–5 weeks of development [28]. The accumulation of ECM material on the midline of the atrial roof indicates the initial stages of formation of the mesenchymal cap. As the pAS starts to grow, the mesenchymal cap becomes clearly visible on its leading edge. The pAS then subsequently grows down (in a posterior-to-anterior direction) as a myocardial sheath from the atrial roof toward the midline of the common AV canal where the two major AV cushions are located (Figure 4C,C’,D,D’ and Figure 5). Fusion of the two major AV cushions results in the separation of the common AV canal into the left AV orifice (in which the mitral valve will develop) and the right AV orifice (in which the tricuspid valve will develop) [19]. The open window between left and right atrium, situated in between the cap on the leading edge and the cushions, is known as the primary (inter) atrial foramen (or foramen primum), which allows for free communication between the left and right atrium (Figure 5A). Fusion of the mesenchymal cap with the (fused) AV cushions will eventually lead to the closure of the primary atrial foramen (Figure 4E,E’). The merging of the cap and the AV cushions results in the formation of a central mass of endocardially derived mesenchyme in which the derivatives of the individual components, at least based on our current knowledge, cannot be distinguished anymore. As the primary foramen is closing, fenestrations start to appear in the (myocardial) upper part of the pAS (Figure 5B). These fenestrations eventually coalesce and form the secondary interatrial foramen (or foramen secundum) [28,52,73]. At this point, the communication between left and right atrium occurs through the secondary foramen. During this process, the secondary atrial septum (sAS or septum secundum) develops within the roof of the right atrium. As this septum (which develops as a thick myocardial ridge) grows into the right atrium, it will start to cover the secondary foramen (Figure 5C,D) without physically fusing with the (lower) part of the primary septum to facilitate the embryonic blood circulation (i.e., before the pulmonary circulation becomes established after birth). After birth, the two septa will typically fuse, a process which has been described to take place in mice around 1–3 months after birth [74]. This fusion also take place in humans. However, in roughly one-third of the human population, the two septa will not fuse, leading to a condition known as patent foramen ovale (PFO), a condition which is sometimes linked to stroke. Failure of the sAS to completely cover the secondary interatrial foramen can lead to a more serious secondary atrial septal defect (sASD or ASD-II), also known as “septum secundum” defect.

## 7. Development of the Mesenchymal Cap and Primary Atrial Septum

**Figure 6:**
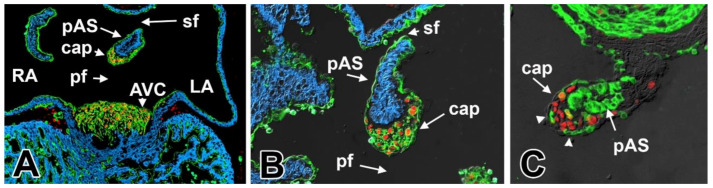
Tissue origin of the mesenchymal cap and primary atrial septum. This figure shows sections of Tie2-cre and ROSA26mT/mG (**A**,**B**) and Mef2c-AHF-cre and ROSA26mT/mG (**C**) hearts at 11.5ED. Cre-induced expression of GFP is shown in green (**A**–**C**), expression of cardiac myosin is visualized in blue (**A**,**B**), and expression of the transcription factor SOX9 is shown in red (**A**–**C**). AVC—atrioventricular cushion; cap—mesenchymal cap; LA—left atrium; pAS—primary atrial septum; pf—primary foramen; RA—right atrium; sf—secondary foramen.

The embryological origins of tissues found in the mouse are nowadays typically studied using cre-lox technology with mouse models that express cre-recombinase under the regulation of tissue-specific promoters. To determine the origin of the tissues of the pASD and mesenchymal cap, we used the Tie2-cre and Nfatc-cre mice (each allowing tracing of the fate of endocardial cells) (Figure 6), and the Mef2c-AHF-cre mouse (to determine the fate of SHF-derived cells). These cre-models were used in combination with the ROSA26^mT/mG^ reporter mouse, in which cre-mediated events lead to the expression of green fluorescent protein (EGFP) (Figure 1, Figure 3 and Figure 6). The results of this study show that the endocardial lining of the cap, as well as the mesenchymal cells within the cap, express EGFP in the Tie2-cre, ROSA26^mT/mG^ and Nfatc-cre, and ROSA26^mT/mG^ mice, indicating that the cap mesenchyme is endocardially derived [5,55] (Figure 6A,B). Furthermore, Mef2c-AHF-cre and ROSA26^mT/mG^ lineage tracing shows that the majority of these cells do not express EGFP, indicating that they are not SHF-derived. It needs to be noted that, based on the presence of some scattered EGFP positive cells, it cannot be excluded that some SHF-derived cells find their way into the mesenchymal cap. Given the fact that the mesenchyme of the DMP and that of the cap are contiguous, this is not too difficult to imagine. The Mef2c-AHF-cre and ROSA26^mT/mG^ lineage tracing experiments also show that, at least part of, the myocardial component of the pAS is derived from the pSHF (Figure 6C). As previously reported, the development of the pAS is intrinsically related to the development of the DMP [14,28,30,70], a structure which, itself, is derived from the pSHF. Taking this into consideration and assuming that, as is the case for the development of the AV cushions, the development of the mesenchymal cap is (at least partly) regulated by myocardially expressed genes, a complex partly pSHF-dependent mechanism for the regulation of the development of the mesenchymal cap should be considered.

## 8. Regulatory Mechanisms Associated with the Development of the Mesenchymal Cap

Insight into the molecular mechanisms involved in the regulation of the development of the various mesenchymal tissues at the AV junction, such as the AV cushions, the DMP, and the epicardium, has significantly evolved over the last 20 years. In particular, the complex regulatory networks that govern the development of the AV cushions have received much attention. It is well established that members of the TGFbeta superfamily of growth factors play a major role in endMT in the cushions [24,75,76,77,78]. The importance of the extracellular matrix (ECM) [79,80,81,82,83] is also well-established, as is the significance of various transcription factors [25,84,85,86,87]. One of the advantages of the study of AV cushion development is that the tissues involved (i.e., AV junctional myocardium and the associated developing cushion material) are relatively easy to isolate and that the cellular behavior of these tissues can be experimentally manipulated using well-established in vitro assays [88,89,90]. Given its size and location, this is unfortunately not the case for this mesenchymal cap. As a result, we rely, at least at this point, mainly on identifying which genes and pathways are associated with the developing cap. With this knowledge, we aim to develop an understanding of how its growth in normal development is regulated and how it might be affected in pathological conditions.

### 8.1. Growth Factors and the Development of the Mesenchymal Cap—TGFbeta and BMP Signaling

**Figure 7:**
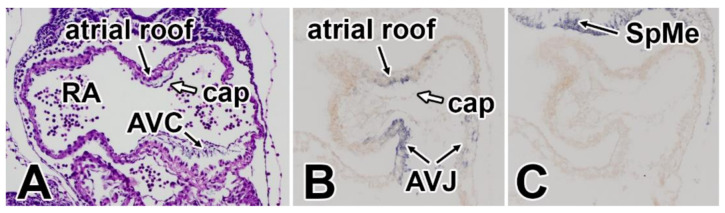
Expression of BMP2 and BMP4. Panel (**A**) shows a H/E staining of a heart at ED9.5. Panel (**B**) shows the expression of BMP2 mRNA in the AV junction and in the myocardial roof of the common atrium adjacent to the developing cap indicated that this growth factor might be involved in the regulation of cap development. Panel (**C**) shows the expression of BMP4, demonstrating the absence of this BMP isoform in AV junction and atrial roof. AVC—atrioventricular cushion; AVJ—atrioventricular junction; cap—mesenchymal cap; RA—right atrium; SpMe—splanchnic mesoderm.

**Figure 8:**
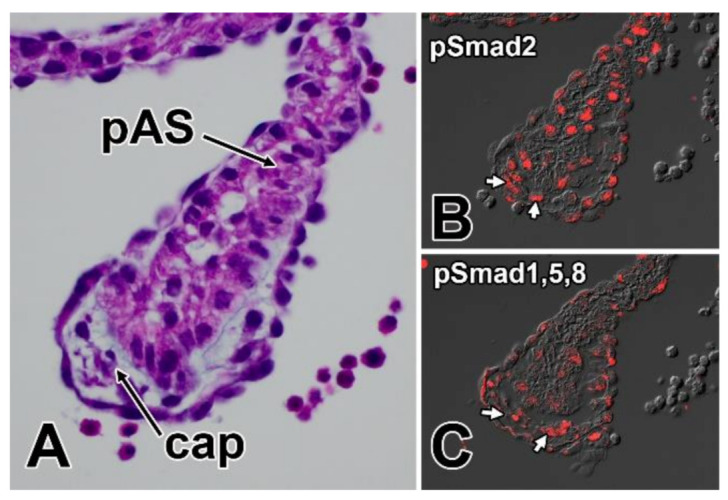
Presence of pSmad2 and pSmad1,5,8 in the mesenchymal cap indicates active signaling through the TGFbeta and BMP pathways. Panel (**A**) shows an H/E staining of the primary atrial septum and mesenchymal cap at ED11.5. The mesenchyme of the cap at this stage stains for pSmad2 (**B**) and pSmad1,5,8 (**C**) indicating active TGFbeta and BMP signaling. cap—mesenchymal cap; pAS—primary atrial septum.

To determine whether growth factor-mediated endMT mechanisms, which have been found to be important in the AV cushions, may also play a role in controlling endMT in the mesenchymal cap, we started by surveying the literature. Given the fact that the mesenchymal cap has received very little recognition as a separate entity and is often (irrespective of what it would have been called) not identified as such, it was not surprising that we were not able to find published papers that specifically focus on the role of growth factor signaling, or any other regulatory mechanisms for that matter, involved in cap development. What we did find, and share in this paper, comes from looking for information in the related literature. In 2002, Gaussin and colleagues published a paper on the role of the BMP receptor ALK3/BMPR1A in heart development [91]. In this paper, images of 10.5–11.5ED mouse hearts are shown that are stained for the presence of multiple genes of interest, including TGFbeta2 and BMP2. While the resolution of these images is limited, the respective panels indicate that both these growth factors, which are also expressed in the AV junctional myocardium, are expressed in the pAS, suggesting that they may be involved in the regulation of endMT in the mesenchymal cap. In a paper on the role of TGFbeta2 in heart development, Bartram and colleagues report on the phenotype of the TGFbeta2 knock-out mouse [92]. They describe how the TGFbeta2 knockout mouse develops a spectrum of different heart defects. In all but one of the 24 inspected mutant mice, the atrial septum had developed properly. In one specimen, however, the pAS had not fused with the AV cushion tissues (which had not developed properly either) but a mesenchymal cap could be seen on its leading edge. In a paper from 2006, Jiao and colleagues investigated the role of TGFbetaR2 in the endocardium by specifically deleting this receptor using the Tie2-cre mouse. While not specifically commenting on the mesenchymal cap, the data presented suggest that deleting this receptor from the endocardium does not have a major impact (if at all) on the initial development of the mesenchymal cap. Revisiting a series of in situ hybridization experiments which we previously published in our 2013 paper on the role of BMP signaling in the pSHF [3], we noted that at ED9.5, BMP2 is expressed in the myocardium adjacent to the developing cap at a stage in which the pAS proper still has to emerge (Figure 7). Following up on the above, and to determine the possible role of TGFbeta and BMP signaling in the development of the cap, we stained for the presence of pSmad2 (indicative for active TGFbeta signaling) and pSmad1,5,8 (indicative for active BMP signaling) (Figure 8). This experiment demonstrated the presence of both sets of signaling intermediates. Combined, these data strongly suggest that TGFbeta and BMP signaling both play a role in the formation of the mesenchymal cap.

### 8.2. Extracellular Matrix and the Mesenchymal Cap—Versican, Link Protein, and Hyaluronan

**Figure 9:**
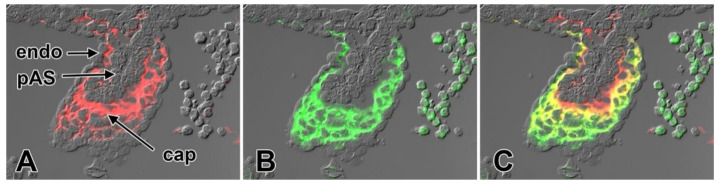
Expression of versican and cartilage link protein 1 in the cap. Immunofluorescent staining for cartilage link protein (CRTL1/HAPLN1) (**A**) and versican (CSPG2) (**B**) show that these ECM proteins are expressed in the mesenchyme of the cap but not in the endocardial lining or in the myocardial part of the pAS. In panel (**C**), the images of A and B are merged. Endo—endocardium; pAS—primary atrial septum.

It is well-established that the ECM plays a significant role in the development of the AV cushions [93,94]. Among the ECM components identified as being critically important in this process are versican (CSPG2) [82,83,95], Cartilage Link Protein 1 (CRTL1 or HAPLN1) [83], and Hyaluronan (HA) [79,80,81]. In particular, CSPG2 and HA (synthesized by Hyaluron synthase 2, HAS2) have been described to be involved in the early stages of cushion formation. While the spatiotemporal expression of these genes in the developing AV cushions is well-documented [83,95], with the exception of CSPG2 [96], little is known about the expression of these genes in the mesenchymal cap. To obtain a better insight into the expression of these ECM members in the developing cap, we stained immunofluorescently and found that CRTL1, CSPG2, and HA (not shown) are expressed in a pattern similar to what has been described in the AV cushions (Figure 9). Whether, and if so how, the development of the cap is affected in any of the knockout models of these ECM components has not been investigated.

### 8.3. Transcription Factors and the Development of the Primary Atrial Septum and the Mesenchymal Cap

**Figure 10:**
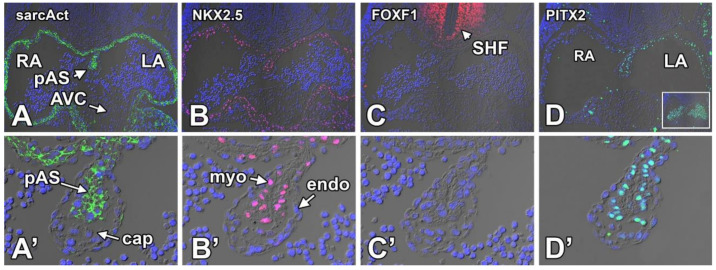
Expression of sarcomeric actin (**A**,**A’**), NKX2.5 (**B**,**B’**), FOXF1 (**C**,**C’**), and PITX2 (**D**,**D’**) in the primary atrial septum and mesenchymal cap at ED11.5. Specifics regarding the observed expression patterns are discussed in the body of the text. AVC—atrioventricular cushion; cap—mesenchymal cap; LA—left atrium; pAS—primary atrial septum; RA—right atrium; myo—myocardial part of the pAS; endo—endocardium.

**Figure 11:**
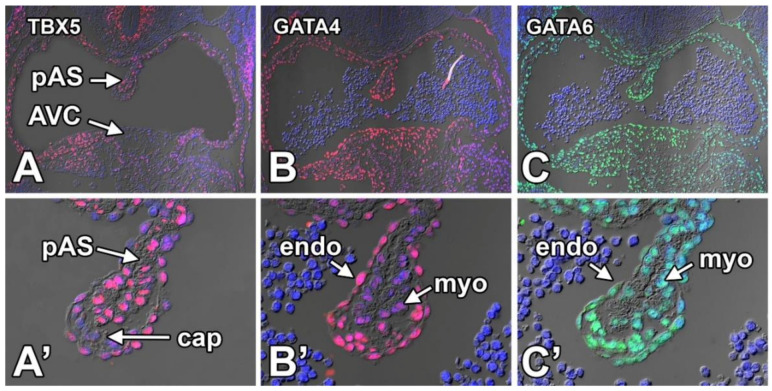
Expression of TBX5 (**A**,**A’**), GATA4 (**B**,**B’**), and GATA6 (**C**,**C’**) in the primary atrial septum and mesenchymal cap at ED11.5. Specifics regarding the observed expression patterns are discussed in the body of the text. AVC—atrioventricular cushion; cap—mesenchymal cap; LA—left atrium; pAS—primary atrial septum; RA—right atrium; myo—myocardial part of the pAS; endo—endocardium.

Atrial septal defects (ASDs) are relatively common congenital heart malformations, observed in approximately 10% of all patients born with congenital heart disease (CHD). An increasing number of genetic mutations have been identified as being associated with these defects. However, not much is known about the specific mechanisms that lead to ASDs. Among the genes found to be associated with ASDs are sarcomeric cardiac alpha-myosin heavy chain protein (MYH6 [97]) and a growing number of transcription factors, including TBX5 [70,98,99], TBX20 [100], SOX9 [101], GATA4 [102,103,104,105], GATA6 [106], and NKX2.5 [102,103]. Additionally, gene deletions in a number of other genes in mice, including members of the Forkhead box transcription factors (FOXF1 and FOXF2) [15] and SOX9 [84,107,108] have been reported to cause ASDs in knockout mouse models. It is important to mention that in humans, mutations in these genes typically lead to sASD (or ASD-II) [98,109,110]. Interestingly, the septal defects seen in mouse models that carry mutations for these genes are often pASDs [70,84,107,108] or not well-defined [111]. This suggests that there may be common components in the pathogenic mechanisms leading to the respective types of ASDs. In an effort to obtain a better insight into how some of these transcription factors might be involved in ASD pathogenesis, we decided to begin with documenting the expression patterns of these candidate genes in pAS and the mesenchymal cap. Some information on the expression of these transcription factors in the atrial septal complex is available in the literature. For instance, the expression of TBX5 in the pAS is well-documented [70,99], as is the expression of some other transcription factors such as NKX2.5 and GATA4 [112]. However, detailed information on the expression of these genes as it relates to the mesenchymal cap is still by and large lacking. To establish how the “ASD-candidate genes” (as well as a few other genes involved in heart development) are expressed in the pAS and the mesenchymal cap, we conducted a series of immunofluorescent studies on mouse embryos at 9.5–11.5ED. We do not claim that the data presented in Figure 10 and Figure 11 are all novel or provide a comprehensive picture of all there is to know about transcription factor expression and pAS/cap development. In future papers, we will build on these data as we explore the roles of selected genes in the pathogenesis of atrial/atrioventricular septal defects, specifically as it relates to the mesenchymal cap. Below, we will described our findings as they relate to the mesenchymal cap at 11.5ED.

#### 8.3.1. Sarcomeric Actin

The staining shown in Figure 10A,A’ is shown as a reference to demonstrate the delineation between the myocardial component of the pAS and the non-myocardial mesenchymal cap and endocardial lining.

#### 8.3.2. NKX2.5

The association between mutations in NKX2.5 and congenital heart disease has been known for over 20 years [113,114]. Mutations in NKX2.5 are linked to sASD, Tetralogy of Fallot (TOF), and conduction abnormalities. Staining performed on ED11.5 mouse hearts shows that NKX2.5 is expressed in virtually all cardiomyocytes, but not in the mesenchyme of the AV cushions, cap, or DMP (Figure 10B,B’). As described in a paper we published in 2007, when the mesenchyme of the DMP undergoes a myocardial differentiation, eventually forming the inferior muscular rim of the atrial septum, the SHF-derived cells from the DMP start to express NKX2.5 [115]. Note that NKX2.5 is not expressed in the pSHF at this stage.

#### 8.3.3. FOXF1

Forkhead box transcription factors have been implicated in the regulation of AV septation by interacting with TBX5 and the Hedgehog signaling pathway [15]. Compound haploinsufficiency for FOXF1a and FOXF2, as well as endothelial-specific knockouts for FOXF1, develop AVSDs [15,116] Immunostaining for FOXF1 at ED11.5 showed that while FOXF1 is expressed in the SHF (Figure 10C), it is not detected in any myocardial structures, the endocardium, or the mesenchymal cap (Figure 10C,C’). Interestingly, the size of the mesenchymal cap in FOXF1A/FOXF2 compound knockout mice was found to be enlarged when compared to control specimens.

#### 8.3.4. PITX2

The homeobox gene PITX2c plays a crucial role in heart development and septation. In mice that lack PITX2c, the normal left/right laterality patterning is disturbed. PITX2c knockout mice develop right atrial isomerism (RAI), a condition in which the left side of the heart develops right-sided characteristics [117]. An AVSD is typically part of the spectrum of cardiac defects observed in mice and humans with RAI. Immunolabeling with an antibody for PITX2 shows expression of PITX2 in the left atrium and in the myocardium of the pAS. PITX2 is not expressed in the mesenchymal cap or the pSHF.

#### 8.3.5. TBX5

Mutations in the T-box transcription factor TBX5 cause Holt–Oram Syndrome (HOS) [98,118], a syndrome characterized by multiple developmental abnormalities. Cardiac defects found in patients with HOS include VSDs, pASDs, and sASDs [119]. In our expression study, focusing on the tissues involved in atrial septation, we found TBX5, as previously reported, predominantly expressed in the pSHF, the atrial myocardium, and the myocardial pAS (Figure 11A,A’). While the vast majority of the mesenchymal cells in the cap do not express TBX5, a few isolated TBX5-positive cells are occasionally observed. TBX5-positive cells are also detected in the endocardial lining of the pAS and mesenchymal cap. As the pSHF also expresses TBX5 and, as shown in Figure 3, the pSHF-derived DMP and the mesenchymal cap are contiguous, it is possible that TBX5 positive cells in the cap are of a pSHF origin.

#### 8.3.6. GATA4

Patients with mutations in GATA4 can develop a number of different congenital heart defects including VSDs and sASDs [105]. GATA4 is strongly expressed throughout the mesenchyme of the cap (Figure 11B,B’) and AV cushions with weaker staining being observed in the myocardium of the pAS and other myocardial structures.

#### 8.3.7. GATA6

The staining observed with the antibody against GATA6 shows very strong expression in the mesenchyme of the cap and AVC. Strong expression is also observed in the myocardium of the pAS and other myocardial structures (Figure 11C,C’). Expression is also observed in the endocardium, but the staining is slightly less intense suggesting a lower level of expression.

#### 8.3.8. SOX9

**Figure 12:**
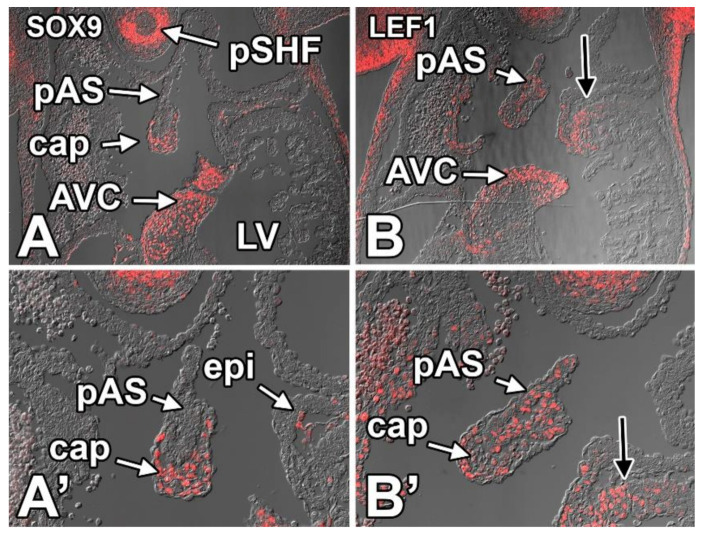
Expression of SOX9 (**A**,**A’**) and LEF1 (**B**,**B’**) in the primary atrial septum and mesenchymal cap at ED11.5. Specifics regarding the observed expression patterns are discussed in the body of the text. AVC—atrioventricular cushion; cap—mesenchymal cap; LA—left atrium; pAS—primary atrial septum; pSHF—posterior second heart field; LV—left ventricle; epi—epicardium.

The significance of the High Mobility Group (HMG) transcription factor SOX9 (SRY-type box 9) in heart development is well-documented. SOX9 is found in the AV cushion mesenchyme where it regulates endMT and the proliferation of the endocardially derived mesenchyme [84,107,108]. Mice in which SOX9 is knocked out develop pASDs [84,107]. Staining for SOX9 shows strong expression in the pSHF and the cap mesenchyme. SOX9 is also expressed in the AV cushions and DMP (not shown in this figure) (Figure 12A,A’). SOX9 is also expressed in epicardial cells at the AV junction and epicardial cells scattered elsewhere on the myocardial surface. No SOX9 expression is seen in the myocardium of the pAS or in any other myocardial structures in the atrium. SOX9 is also not seen in the endocardium.

#### 8.3.9. LEF1

Lymphoid enhancer binding factor (LEF1) is a downstream target of WNT/beta-catenin signaling and its expression in cells indicates activation of this signaling pathway. LEF1 expression was observed, albeit scattered, in the pSHF, the pAS, and the mesenchymal cap, suggesting that WNT/beta-catenin signaling is playing a role in the development of all these structures. LEF1 expression is also observed in the myocardial “shoulders” of the left and right ventricles (black arrows) (Figure 12B,B’).

### 8.4. Conclusions

The pAS and the associated mesenchymal cap express a variety of genes that are known to be expressed and involved in the development of other components of the atrioventricular septal complex. Whether the development of the cap is regulated in ways similar to that described for the AV cushions and the DMP remains to be elucidated. Retrospective studies (where possible) on the consequences of knockout models for candidate genes here described and other genes of interest as well as new studies focusing on the development of the mesenchymal cap may provide new insights into whether perturbation of cap formation may be a factor in the pathogenesis of congenital heart defects.

## 9. Discussion and Future Directions

For many years, proper development of the AV cushions was seen as the most critical event in AV valvuloseptal morphogenesis and considered crucial for correct formation of the septal structures at the AV junction. Cushions hypoplasia and/or failure of the cushions to fuse was for decades seen as the primary mechanism leading to AVSDs. Because of that, these congenital defects were (and still are) typically referred to as “endocardial cushion defects”. However, as pointed out by Professor Anderson in a paper from 2010 [66], AVSDs can be found in animal models in which the cushions are actually larger than normal and in settings where the cushions actually have fused. Studies conducted over the last 10 years have demonstrated that interfering with the development of the SHF-derived DMP, without affecting the initial development of the AV cushions, can generate AVSDs. This led to a paradigm shift in the understanding of the pathogenesis of AVSD as it showed that the development of the DMP, rather than that of the AV cushions, could be the most critical step in AV septal development. The work presented in this paper focuses on the third, by and large historically ignored, component of the AV mesenchymal complex, i.e., the mesenchymal cap on the leading edge of the primary atrial septum. Compared to what is known about the AV valves and the DMP, understanding of the mechanisms that drive the development of the mesenchymal cap and an appreciation of its role in AV septation are still in their infancy. In this contribution, we have presented data on how the cap develops and showed results of studies that provide insight on how a number of signaling mechanisms, transcription factors, and extracellular matrix components might be involved in controlling the development of the cap. In ongoing studies, we are building on these (preliminary) results by specifically focusing on cap development in mouse models in which candidate genes believed to be important for the development of the AV mesenchymal complex are deleted in a tissue-specific manner. These studies will provide more information of how the cap is forming, but also on its the specific role in AV septation; we believe that they will reveal that the cap is playing a significant role in AV septation.

## 10. Methods

### 10.1. Mice

Generation and use of the cre-recombinase mouse models used for cell fate tracing (Mef2c-AHF-cre, Tie2-cre, and Nfatc1-cre) have been described previously [3,12,120,121]. The cre-mice were used in combination with the B6.129(Cg)-Gt(ROSA)26Sor^tm4(ACTB-tdTomato,dTomatLuo/J^ (R26^mT/mG^ reporter mouse (Jackson Laboratory; stock no 007676). Staging and hematoxylin/eosin staining were performed as previously described [122]. All experiments using animals were approved by the MUSC Institutional Animal Care and Use Committee (IACUC) and complied with federal and institutional guidelines. Tissue processing and hematoxylin/eosin staining was performed as previously described.

### 10.2. Immunolabeling

Primary antibodies: Myosin Heavy Chain (MF20; DSHB), Sarcomeric Actin (Sigma #A2172), pSMAD1,5,8 (Cell Signaling #13820), pSMAD2 (Cell Signaling #3108), VERSICAN, CRTL1/HAPLN1 (DSHB #9/30/8-A-4-c), GATA4 (Santa Cruz #sc-1237), GATA6 (R&D #AF1700), TBX5 (R&D #AF5918), NKX2.5 (R&D #AF2444), SOX9 (Novus #NBP1-8555), FoxF1 (R&D #AF4798), and EGFP (Aves Labs Inc. #GFP-1020). Secondary antibodies were obtained from Jackson Immunoresearch and Invitrogen. Nuclei were detected using DAPI (Invitrogen, Carlsbad, CA, USA; Slowfade Gold Antifade Reagent with DAPI; catnr S36938). Fluorescent staining was visualized using a Zeiss AxioImager II microscope.
